# Integrative analysis of mitochondrial metabolic reprogramming in early-stage colon and liver cancer

**DOI:** 10.3389/fonc.2023.1218735

**Published:** 2023-08-24

**Authors:** Yeongmin Kim, So-Yeon Shin, Jihun Jeung, Yumin Kim, Yun-Won Kang, Sunjae Lee, Chang-Myung Oh

**Affiliations:** ^1^ Department of Biomedical Science and Engineering, Gwangju Institute of Science and Technology, Gwangju, Republic of Korea; ^2^ Department of School of Life Sciences, Gwangju Institute of Science and Technology, Gwangju, Republic of Korea

**Keywords:** colon cancer, hepatocellular carcinoma, mitochondria, metabolic reprogramming, 3-hydroxy-3-methylglutaryl-CoA synthase 2 (HMGCS2)

## Abstract

Gastrointestinal malignancies, including colon adenocarcinoma (COAD) and liver hepatocellular carcinoma (LIHC), remain leading causes of cancer-related deaths worldwide. To better understand the underlying mechanisms of these cancers and identify potential therapeutic targets, we analyzed publicly accessible Cancer Genome Atlas datasets of COAD and LIHC. Our analysis revealed that differentially expressed genes (DEGs) during early tumorigenesis were associated with cell cycle regulation. Additionally, genes related to lipid metabolism were significantly enriched in both COAD and LIHC, suggesting a crucial role for dysregulated lipid metabolism in their development and progression. We also identified a subset of DEGs associated with mitochondrial function and structure, including upregulated genes involved in mitochondrial protein import and respiratory complex assembly. Further, we identified mitochondrial 3-hydroxy-3-methylglutaryl-CoA synthase (*HMGCS2*) as a crucial regulator of cancer cell metabolism. Using a genome-scale metabolic model, we demonstrated that *HMGCS2* suppression increased glycolysis, lipid biosynthesis, and elongation while decreasing fatty acid oxidation in colon cancer cells. Our study highlights the potential contribution of dysregulated lipid metabolism, including ketogenesis, to COAD and LIHC development and progression and identifies potential therapeutic targets for these malignancies.

## 1 Introduction

Cancer is the leading cause of death worldwide, accounting for 19.3 million new cases and nearly 10.0 million deaths in 2020 (1). The socioeconomic burden of cancer has dramatically increased. In the United States, the economic burden on patients was higher than $21.09 billion in 2019 (2). Although lung cancer is the major cause of cancer-related deaths (18%), gastrointestinal (GI) colorectal and liver cancers (9.4% and 8.3%, respectively) are the second most common causes (1). Despite substantial advances in cancer research in recent decades, the survival rate for these cancers remains remarkably low. Colorectal cancer has a 5-year overall survival rate of approximately 60% (14% of patients with distant metastasis) (3), and liver cancer has a 5-year survival rate of approximately 20% (3% of patients with distant metastasis) (4). Although they occur in different organs, these two cancers share common underlying mechanisms such as inflammation, oxidative stress, and alterations in signaling pathways, which promote their development and progression. Therefore, studying the common mechanisms of these two cancers can provide valuable insights into the fundamental processes of cancer biology and have important clinical implications (5).

In 1930, Warburg discovered alterations in cancer cell metabolism, indicating increased aerobic glycolysis with a high rate of lactate production for biomass synthesis and rapid ATP production (6). Reprogramming of cellular metabolism has been identified as a hallmark of cancer (7) and cancer cell metabolism has been recognized as a promising treatment target (8). Intriguingly, epidemiological studies have also revealed that chronic metabolic stress, such as obesity and diabetes mellitus, is associated with the development of these two GI cancers with the highest mortality rate (9–12). However, little is known about the role of metabolic dysregulation in the early stages of tumorigenesis.

Previously, the Warburg effect was considered a compensatory mechanism for mitochondrial dysfunction in cancer cells (13). However, recently, the mitochondria, which are critical players in cellular energy metabolism, were found to play essential roles in promoting cancer cell growth and tumorigenesis (13, 14). Mitochondrial dysregulation can contribute to the development and progression of cancer by altering energy metabolism, promoting oxidative stress and inflammation, and affecting cellular signaling pathways (15).

Therefore, elucidating the complex interplay between mitochondrial function and cancer biology is critical for developing effective therapies. In this study, we performed a comparative analysis of genetic signatures from normal and GI cancer tissues obtained from The Cancer Genome Atlas (TCGA) to gain insight into the pathogenesis of colon adenocarcinoma (COAD) and hepatocellular carcinoma (LIHC) (16). Our analysis revealed that mitochondrial 3-hydroxy-3-methylglutaryl-CoA synthase (HMGCS2), a key enzyme in ketogenesis and member of the HMG-CoA protein family, is a crucial regulator of cancer cell metabolism (17). Specifically, we found that *HMGCS2* expression was downregulated in both COAD and LIHC tissues compared to that in normal tissues. Furthermore, using a genome-scale metabolic model (GSM), we showed that *HMGCS2* suppression increased glycolysis, lipid biosynthesis and elongation, and decreased fatty acid oxidation (FAO). Finally, *in vitro* experiments using cancer cell lines provided further evidence to support the role of HMGCS2 in cancer cell metabolism. Collectively, our findings suggest that dysregulated lipid metabolism, including decreased ketogenesis due to *HMGCS2* suppression, is a potential therapeutic target for treating GI malignancies.

## 2 Materials and methods

### 2.1 Colon adenocarcinoma and lung adenocarcinoma data

The RNA-seq data for COAD and LIHC were downloaded from TCGA portal (18). The data type derived from TCGA was used only for STAR-Counts. We obtained 437 COAD and 424 LIHC RNA-seq datasets. To identify metabolic alterations during the early stages, stage I cancer data were selected by comparison with the metadata derived from TCGA. Finally, we obtained 39 normal and 62 tumor samples from COAD, and 50 normal and 171 tumor samples from LIHC.

### 2.2 RNA-seq analysis

To ensure data quality, we filtered the STAR counts by removing those with average counts of less than one in all patients. We then applied DESeq2 in Bioconductor (19) to normalize the filtered count data and extract differentially expressed genes (DEGs) from normal and tumor tissues with an adjusted p-value cutoff of 0.01. To visualize the DEGs, we used a cutoff of |log2foldchange (log_2_FC)| > 0.58 and converted any genes with p-adjust value (p_adj_) or Log_2_FC as NA to “1” to prevent undetectable error. The DEGs were displayed using Enhanced Volcano in Bioconductor (20), where the gray dots represented “non-DEGs,” red dots represented “log_¬2_FC > 0.58 and p_adj_ <0.01,” and blue dots represented “log_2_FC < -0.58 and p_adj_ <0.01”.

### 2.3 Principal component analysis plot generation

Each gene in the normal and tumor tissues in COAD and LIHC contained numerous dimensions. To visualize the genes, dimensionality reduction was performed using principal component analysis (PCA) and the results were visualized using ggplot2 in R (21). The PCA plot visualizes PC1 on the x-axis and PC2 on the y-axis, and the normal and tumor groups are represented by ellipses.

### 2.4 Gene ontology enrichment analysis and gene set enrichment analysis

To comprehensively understand the functions of the DEGs, we conducted a Gene Ontology (GO) enrichment analysis using ClusterProfiler in Bioconductor (22). Specifically, we used a p-adjusted value cutoff of 0.01 for genes with a log_2_FC > 0.58 and log_2_FC < -0.58 to indicate upregulated and downregulated genes, respectively. To confirm the metabolic process alterations in the early stages of tumorigenesis, we focused only on biological process (BP) terms that indicate cellular or physiological effects. The results of the GO enrichment analysis are displayed as a heatmap with -log10 p-values, where the upregulated gene set is depicted in red, and the downregulated gene set is depicted in blue. After conducting the GO analysis, we visualized the results using a heatmap. A heat map was generated using the pheatmap function in Bioconductor, which showed the expression levels of the identified genes (23).

To further investigate the metabolic processes involved in COAD and LIHC, we utilized the Gene Set Enrichment Analysis (GSEA) tool provided by ClusterProfiler in Bioconductor (24). The analysis was conducted using a p-value cutoff of 0.05, and only BP (biological process) gene set terms were considered to compare metabolic processes in both cancers. The GSEA results are presented using an enrichment plot in Bioconductor (25) and include the normalized enrichment score (NES) and corresponding p-value.

### 2.5 Genome-scale metabolic model analysis

In this study, we performed constraint-based simulations using two genome-scale metabolic models (GSMs) to elucidate the functional role of HMGCS2 in cancer metabolism. Specifically, we utilized the colon cancer model (26) and the iHepatocytes2322 curated liver model (27) and conducted simulations using the COBRA Toolbox v.3.0[28] and the method of minimization of metabolic adjustment (28). We generated *HMGCS2* knock-out colon models by limiting the lower bounds of the HMGCS2-related reactions (HMR1437, HMR4604, and HMR1573) to nine, while the *HMGCS2*-overexpressed colon models had upper bounds of 4000 for these three reactions. Similarly, *HMGCS2* knock-out liver models were derived from iHepatocytes2322 by limiting the lower bounds of HMGCS2-related five reactions (HMR1437, HMR4604, HMR1573, HMR0027, and HMR0030) to 0, while *HMGCS2*-overexpressed liver models had a lower bound of 2000 and an upper bound of 4000 for these five reactions.

To investigate the functional role of HMGCS2 in cancer metabolism, we observed changes in reaction flux by genetically altering HMGCS2. Specifically, we defined reactions whose flux decreased in *HMGCS2* knock-out models and increased in *HMGCS*2 overexpression models as “flux decreasing” reactions, while reactions whose flux increased in *HMGCS2* knock-out models and decreased in *HMGCS2* overexpression models were defined as “flux increasing” reactions. We then counted the number of flux-increasing and decreasing reactions per subsystem and categorized these numbers by the total number of reactions in each subsystem to summarize flux changes.

Next, we analyzed the effects of gene perturbation of HMGCS2 in glycolysis and lipid metabolism in colon and liver models. Specifically, we calculated flux changes by subtracting the fluxes of the original models from those of the perturbation models and considered flux changes higher than 10% of the original flux with positive and negative signs as “up-regulated” and “down-regulated,” respectively. Reactions whose changes were neither up- nor down-regulated were assigned as “no change,” while reactions that were unidentified in the model were indicated as “unidentified”.

### 2.6 Measurement of oxygen consumption rate and extracellular acidification rate

Colon cancer (Caco-2) cells, derived from human colorectal adenocarcinoma, were procured from ATCC and maintained in Dulbecco’s Modified Eagle’s Medium supplemented with 10% fetal bovine serum and 1% penicillin-streptomycin-amphotericin B at 37°C with 5% CO_2_. To target HMGCS2 [NM_001166107.1 and NM_005518.3], siRNA sequences were purchased from Bioneer (Korea), and Lipofectamine RNAiMAX (Thermo Fisher Scientific, Inc., MA, USA) was used to transfect the siRNA according to the manufacturer’s instructions.

To measure the Oxygen Consumption Rate (OCR) and Extracellular Acidification Rate (ECAR) of Caco-2 monolayers, we employed a Seahorse XFp Extracellular Flux Analyzer (Agilent Technologies, Santa Clara, CA, USA). The Seahorse XFp Sensor Cartridge was pre-hydrated with XFp Callibrant solution one day prior to the test and incubated overnight at 37°C in a CO_2_-free incubator to eliminate CO_2_, which could interfere with pH-sensitive measurements. Subsequently, Caco-2 cells were seeded onto XFp Miniplates at a density of 2×10^4^ cells/well and allowed to settle overnight. On the day of the assay, the complete growth medium was replaced with 180 ul/well of XF assay medium, which was maintained at 37°C in a non-CO_2_ incubator for 1 h to allow pre-equilibration with the XF assay medium. We then analyzed the mitochondrial function of the cells by sequentially injecting oligomycin (1 µM), carbonyl cyanide-4 (trifluoromethoxy) phenylhydrazone (FCCP, 0.5 µM), and a mix of rotenone and antimycin A. Finally, OCR and ECAR values were normalized using cellular protein content.

## 3 Results

### 3.1 Identifying common and unique transcriptomic signatures of colon cancer and hepatocellular carcinoma

The present study aimed to identify common genetic foundations and related signaling pathways in GI malignancies. We extensively analyzed the publicly accessible TCGA database, focusing on the COAD and LIHC datasets comprising 437 and 424 samples, respectively. To investigate the metabolic changes in early tumorigenesis, we used only Stage I cancer data for further analysis, resulting in 39 normal samples and 62 tumor samples for COAD, and 50 normal samples and 171 tumor samples for LIHC.

**Figure 1:**
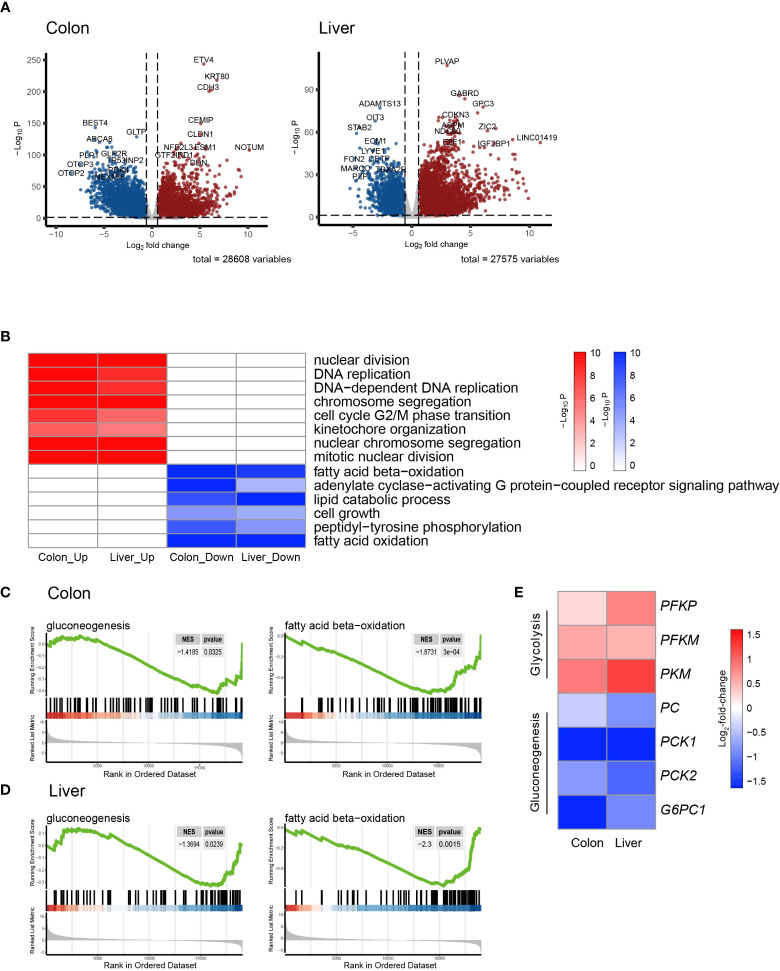
Transcriptomic signatures of colon cancer and hepatocellular Carcinoma. **(A)** Volcano plot showing the differentially expressed genes (DEGs) in colon adenocarcinoma (COAD) and hepatocellular carcinoma (LIHC) compared to normal tissue. **(B)** Heatmap of GSEA enriched pathways from the common DEGs of COAD and LIHC. **(C)** Enrichment plots related to glycose and lipid metabolism in COAD. **(D)** Enrichment plots related to glycose and lipid metabolism in LIHC. **(E)** Heatmap of gene sets related glycolysis and gluconeogenesis in COAD and LIHC. DEG, differentially expressed gene; COAD, colon adenocarcinoma; LIHC, liver hepatocellular carcinoma; GSEA, gene set enrichment analysis.

As is demonstrated in **Supplementary Figure 1A**, the PCA plot clearly displays distinct elliptical clusters that effectively separated the normal and tumor samples. This supports the notion that the expression profiles of GI systems change substantially due to tumorigenesis. Using a list of DEGs, we generated volcano plots (**Figure 1A**) to identify significant differences in gene expression profiles between normal and cancer tissues. We found 7837 and 8767 up-regulated genes and 7232 and 3642 down-regulated genes in the colon and liver tissues, respectively. **Tables 1**, **2** show the top ten upregulated and downregulated DEGs in both COAD and LIHC tissues based on p-values. In COAD, ETS variant transcription factor 4 (*ETV4*), keratin 80 (*KRT80*), and forkhead box Q1 (*FOXQ1*) were the top three upregulated genes, whereas estrophin 4 (*BEST4*), glycolipid transfer protein (*GLTP*), and carbonic anhydrase 7 (*CA7*) were the top three downregulated genes. Similarly, in LIHC, plasmalemma vesicle-associated protein (*PLVAP*), collagen type XV alpha 1 chain (*COL15A1*), and gamma-aminobutyric acid type A receptor subunit delta (*GABRD*) were the top three upregulated genes, whereas ADAM metallopeptidase with thrombospondin type 1 motif 13 (*ADAMTS13*), oncoprotein induced transcript 3 (*OIT3*), and stabilin 2 (*STAB2*) were the top three downregulated genes.

**Table 1:** List of top ten up- and down-regulated differentially expressed genes between colon cancer and normal tissue.

	Gene	*p*-Value	*p*-Adj	Log2FC	Description
Up	ETV4	6.69E-249	1.92E-244	5.388356	ETS variant transcription factor 4
	KRT80	4.39E-223	6.29E-219	6.767	keratin 80
	FOXQ1	2.57E-207	2.46E-203	6.185771	forkhead box Q1
	CDH3	3.61E-205	2.59E-201	5.942379	cadherin 3
	CEMIP	5.39E-154	3.09E-150	5.079765	cell migration inducing hyaluronidase 1
	CLDN1	4.40E-137	1.80E-133	5.050994	claudin 1
	AJUBA	1.66E-122	3.97E-119	3.008937	ajuba LIM protein
	CASC19	1.92E-122	4.23E-119	4.896743	prostate cancer associated transcript 2
	ESM1	2.16E-116	4.29E-113	5.556778	endothelial cell specific molecule 1
	NFE2L3	2.24E-116	4.29E-113	2.753282	NFE2 like bZIP transcription factor 3
Down	BEST4	9.91E-148	4.73E-144	-5.91417	bestrophin 4
	GLTP	8.82E-133	3.16E-129	-1.59429	glycolipid transfer protein
	CA7	9.20E-129	2.93E-125	-5.9989	carbonic anhydrase 7
	ABCA8	2.90E-124	8.33E-121	-5.48495	ATP binding cassette subfamily A member 8
	TMEM100	7.48E-124	1.95E-120	-4.4167	transmembrane protein 100
	SLC25A34	2.61E-116	4.68E-113	-4.19548	solute carrier family 25 member 34
	FAM135B	7.26E-116	1.22E-112	-4.71194	family with sequence similarity 135 member B
	MAMDC2	1.54E-108	1.84E-105	-5.73998	MAM domain containing 2
	PCSK2	2.00E-108	2.29E-105	-6.76073	proprotein convertase subtilisin/kexin type 2
	GLP2R	4.44E-106	4.72E-103	-3.9582	glucagon like peptide 2 receptor

**Table 2:** List of top ten up- and down-regulated differentially expressed genes in colon cancer and normal tissue.

	Gene	*p*-Value	*p*-Adj	Log2FC	Description
Up	PLVAP	2.52E-111	6.97E-107	3.002353	plasmalemma vesicle associated protein
	COL15A1	1.26E-90	1.74E-86	4.023823	collagen type XV alpha 1 chain
	GABRD	3.05E-88	2.81E-84	4.507174	gamma-aminobutyric acid type A receptor subunit delta
	GPC3	2.33E-82	1.61E-78	6.061433	glypican 3
	THBS4	4.98E-78	2.29E-74	5.593584	thrombospondin 4
	DIPK2B	7.57E-75	2.99E-71	2.301232	divergent protein kinase domain 2B
	SLC26A6	1.27E-74	4.39E-71	2.598325	solute carrier family 26 member 6
	CDKN3	2.48E-74	7.62E-71	3.725304	cyclin dependent kinase inhibitor 3
	FOXM1	1.17E-72	3.23E-69	3.231552	forkhead box M1
	NUF2	2.19E-72	5.50E-69	3.854367	NUF2 component of NDC80 kinetochore complex
Down	ADAMTS13	1.38E-81	7.63E-78	-2.70486	ADAM metallopeptidase with thrombospondin type 1 motif 13
	OIT3	6.41E-72	1.26E-68	-3.10719	oncoprotein induced transcript 3
	STAB2	4.16E-67	4.79E-64	-4.43614	stabilin 2
	ECM1	1.75E-57	1.10E-54	-3.08879	extracellular matrix protein 1
	MAP2K1	2.27E-55	1.08E-52	-1.33004	mitogen-activated protein kinase kinase 1
	CCL23	4.26E-55	1.96E-52	-2.87074	C-C motif chemokine ligand 23
	BMPER	4.82E-52	1.73E-49	-4.41371	BMP binding endothelial regulator
	TRIB1	1.33E-51	4.61E-49	-1.99208	tribbles pseudokinase 1
	PTH1R	1.68E-50	5.00E-48	-3.26794	parathyroid hormone 1 receptor
	LYVE1	5.70E-50	1.56E-47	-3.28161	lymphatic vessel endothelial hyaluronan receptor 1

To gain further insight into the metabolic pathways that were enriched during the early stages of tumorigenesis, we conducted a pathway enrichment analysis using GSEA. As shown in **Figure 1B** and **Supplemental Figure 1B**, the heatmap displays the enriched pathways in cancer and normal tissues. The analysis revealed that the genes differentially expressed during early tumorigenesis are associated with various aspects of cell cycle regulation. Notably, genes involved in “DNA replication,” “mitotic nuclear division,” and “cell cycle G2/M phase transition” were found to be positively enriched in both COAD and LIHC. Furthermore, the results indicate that genes related to lipid metabolism were significantly enriched in COAD and LIHC. Specifically, “fatty acid beta-oxidation (FAO)” and “cellular lipid catabolic process” were found to be negatively associated with early tumorigenesis in both cancer types (**Figures 1B–D**). These findings suggested that dysregulated lipid metabolism is crucial in the development and progression of COAD and LIHC.

To assess glucose metabolism in both COAD and LIHC groups, we compared the gene expression of key irreversible enzymes involved in regulating glycolysis and gluconeogenesis (**Figure 1E**). The major rate-limiting enzymes in glycolysis, including phosphofructokinase homologs (*PFKP* and *PFKM*) and pyruvate kinase (*PKM*), which were significantly increased in both COAD and LIHC. Conversely, the levels of key enzymes related to gluconeogenesis, such as pyruvate kinase (*PC*), phosphoenolpyruvate carboxykinase (*PCK1* and *PCK2*), and glucose-6-phosphatase (*G6PC1* and *G6PC2*), were significantly decreased. These findings were consistent with the expected alterations in glucose metabolism in COAD and LIHC, commonly known as the Warburg effect (29), suggesting a shift towards increased glucose uptake and utilization through glycolysis in these malignancies.

### 3.2 Comparison of transcriptomic signatures for mitochondrial energy metabolism in colon cancer and hepatocellular carcinoma

**Figure 2:**
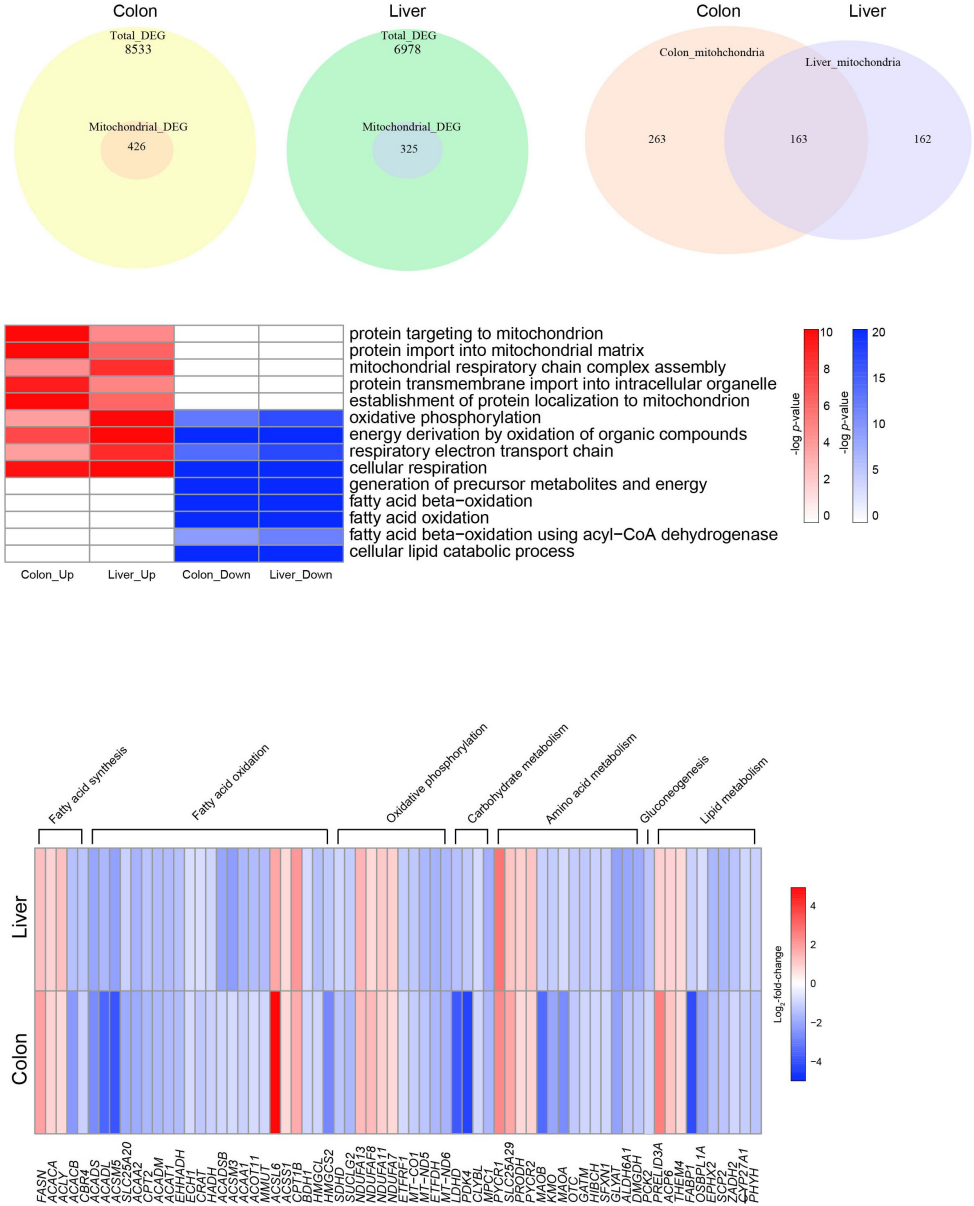
Altered mitochondrial energy metabolism in colon cancer and hepatocellular carcinoma. **(A)** Venn diagrams indicating the numbers of total DEGs and mitochondrial DEGs in COAD and LIHC. **(B)** Venn diagrams indicating overlapping genes between mitochondrial DEGs of COAD and LIHC. **(C)** Heatmap of GSEA enriched pathways from the common mitochondrial DEGS of COAD and LIHC. **(D)** Heatmap of DEGs related glucose and lipid metabolism. DEG, differentially expressed gene; COAD, colon adenocarcinoma; LIHC, liver hepatocellular carcinoma.

Mitochondria are key organelles in cellular energy metabolism, as they serve as the primary sites for oxidative phosphorylation (OXPHOS) and FAO, and for ATP production (30). When analyzing the DEGs in COAD and LIHC, we identified a specific subset of 426 and 325 genes, respectively, that were significantly linked to mitochondrial function and structure (31) (**Figure 2A**). Notably, among the mitochondrial genes identified, 164 were common DEGs between the two cancers (**Figure 2B**).

As shown in **Figure 2C** and **Supplementary Figure 2**, our results demonstrate the enrichment of mitochondrial genes based on the DEGs identified between cancer and normal tissue samples. Interestingly, we observed an upregulation in genes involved in “mitochondrial protein import” and “mitochondrial respiratory complex assembly,” which are critical components of mitochondrial biogenesis and energy generation (32, 33), in both COAD and LIHC. Conversely, we noted a downregulation of genes related to “FAO” and “lipid catabolic process.” Our findings suggest a potential shift in the metabolic profile of GI cancers towards an increased reliance on mitochondrial biogenesis and a decreased dependence on lipid metabolism.

The heat map displayed in **Figure 2D** shows the common DEGs involved in mitochondria-related metabolism in both COAD and LIHC. Our analysis revealed a significant increase in the expression of genes associated with fatty acid synthesis, whereas most genes related to FAO were downregulated. Furthermore, we observed a decrease in several genes involved in tryptophan metabolism, including kynurenine 3-monooxygenase (KMO) (34) and monoamine oxidase A (MAOA) (35). Additionally, we noted a decrease in the expression of the succinate dehydrogenase complex subunit D (SDHD) gene, which encodes a subunit of the mitochondrial enzyme responsible for succinate oxidation and is a well-known tumor suppressor (36). These results provide important insights into the altered metabolic pathways in GI cancers, which may contribute to their development and progression.

### 3.3 HMGCS2: a possible key determinant of energy metabolism in GI malignancies

**Figure 3:**
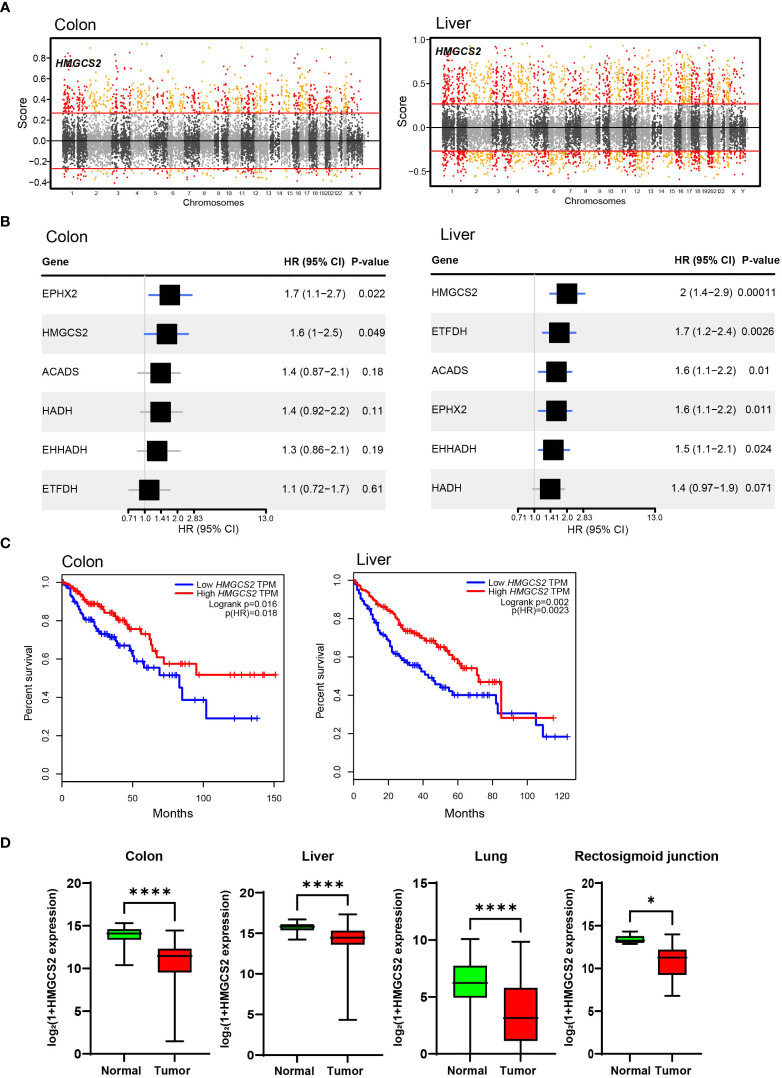
Significance of HMGCS2 as a Prognostic Marker for GI Malignancies. **(A)** Manhattan plot for module: fatty acid oxidation in colon and liver. **(B)** Overall survival according to *HMGCS2* expression in COAD and LIHC. **(C)** *HMGCS2* expression in colon, liver, lung, and rectosigmoid junction cancer. COAD, colon adenocarcinoma; LIHC, liver hepatocellular carcinoma. *p<0.05; ****p<0.0001.

To identify crucial candidates that regulate energy metabolism in GI malignancies, we conducted a correlation network analysis using the GeneBridge toolkit (37). This newly developed bioinformatics tool allows the imputation of gene functions and biological connectivity using large-scale multispecies expression datasets (37). The analysis revealed that 285 genes in COAD and 2399 genes, including 3-Hydroxy-3-Methylglutaryl-CoA Synthase 2 (*HMGCS2*), were associated with “fatty acid oxidation” (GO:0006635) (**Figure 3A**). Among these genes, 25 genes including 3-Hydroxy-3-Methylglutaryl-CoA Synthase 2 (*HMGCS2*) are common mitochondrial genes between COAD and LIHC. To identify crucial mitochondrial genes associated with GI malignancies, we calculated the hazard ratio (HR) for each gene’s related all-cause mortality in COAD and LIHC. **Figure 3B** displays the HR of common mitochondrial genes, with *HMGCS2* being one of the most highly expressed HR genes in both cancers. Patients with low *HMGCS2* expression had higher HR than those with high HMGCS2 expression in both malignancies.

Then, we performed survival analyses of cancer patients based on the expressions of DEGs that are commonly observed in COAD and LIHC using the GEPIA tool (38). By analyzing common DEGs, we identified a set of 25 genes that were particularly linked to FAO. Moreover, our investigation revealed 6 genes that have a noteworthy impact on the survival of patients with cancer. Of these 6 genes, HMGCS2 was the only gene that displayed a statistically significant difference in the overall survival rates of patients with both COAD and LIHC (**Figure 3C**; **Supplementary Figure 3**). Notably, the expression of HMGCS2 was found to be considerably reduced in lung cancer and rectosigmoid junction cancer, and in COAD and LIHC, compared to normal tissues (**Figure 3D**). In addition, HMGCS2 expression was also found to be significantly lower in colon and liver cancer, as shown in **Supplementary Figure 4**, where we analyzed public cancer datasets for colon and liver cancer. These results indicated that HMGCS2 may play a critical role in the pathogenesis of GI malignancies.

### 3.4 Predictive modeling of HMGCS2-driven metabolic flux in GI malignancies

**Figure 4:**
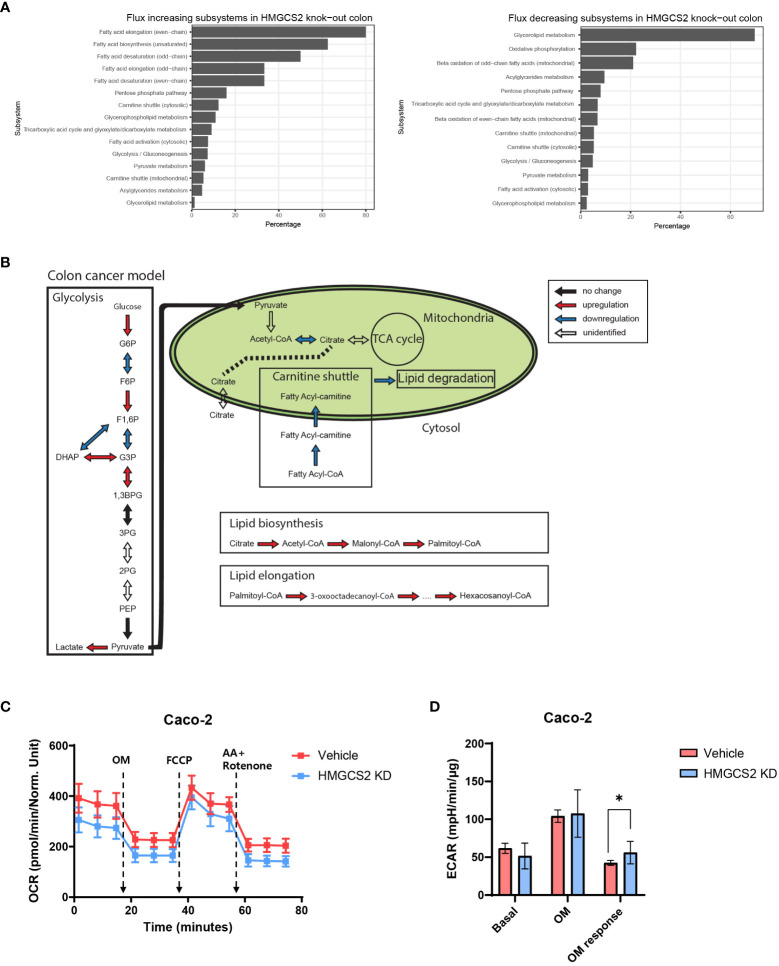
Prediction of HMGCS2-driven metabolic flux. **(A)** Bar plots of predicted increasing and decreasing subsystems according to *HMGCS2* knock-out in colon cancer using genome-scale metabolic model. **(B)** Schematic overview of the metabolic flux according to *HMGCS2* knock-out in colon cancer in the genome-scale metabolic model. **(C)** Real-time assessment of oxygen-consumption rate in control (Vehicle) and *HMGCS2* knockdown (KD) Caco-2 Cells: basal and mitochondrial stress conditions with oligomycin, FCCP, and rotenone plus antimycin. **(D)** Normalized extracellular acidification rate in Vehicle and *HMGCS2* KD cells. OM, oligomycin. *p<0.05.

To gain further insight into the metabolic functions of HMGCS2 in GI malignancies, we conducted genome-scale metabolic simulations using the COAD and LIHC models. In the COAD model, the suppression of *HMGCS2* led to a significant increase in the fluxes of over half of the reactions in the fatty acid synthesis subsystems (i.e., fatty acid biosynthesis and elongation), whereas the fluxes in the fatty acid degradation subsystems (i.e., fatty acid destruction, beta-oxidation, and mitochondrial carnitine shuttle) were significantly reduced (**Figures 4A, B**; **Supplementary Figure 2**). Furthermore, HMGCS2 inhibition resulted in a notable upregulation in the flux of glycolysis subsystems and downregulation in the flux of oxidative phosphorylation. Remarkably, the suppression of *HMGCS2* resulted in similar changes in metabolic flux in a normal liver tissue model (**Supplementary Figure 5**). However, in the LIHC model, suppression of HMGCS2 did not cause significant changes in metabolic flux prediction.

To further investigate the role of HMGCS2 in energy metabolism in cancer cells, we measured oxidative phosphorylation and glycolysis using a Seahorse extracellular flux analyzer (39). Our investigation focused on human Caco-2 cells and aimed to explore the effects of HMGCS2 inhibition on these metabolic pathways. *HMGCS2* knockdown resulted in a discernible decrease in the OCR of Caco-2 cells, suggesting decreased oxidative phosphorylation (**Figure 4C**). We also noticed a corresponding increase in the ECAR in these cells, indicating enhanced glycolysis (**Figure 4D**; **Supplementary Figure 6**). These results support the notion that the inhibitory effects of HGMCS2 alter the metabolic flux, which is in line with the predictions made by our model.

## 4 Discussion

In this study, we aimed to identify common genetic profiles and related signaling pathways in gastrointestinal malignancies, specifically COAD and LIHC. Transcriptomic analysis using TCGA database revealed that the expression profiles of GI systems resulting from tumorigenesis effectively separated normal and cancer tissues, as was evidenced by distinct elliptical clusters in the PCA plot. From DEG analysis, we identified significant changes in gene expression between normal and cancerous tissues. In COAD, *ETV4* was the most highly upregulated gene compared to normal tissues. Recently, this transcription factor was shown to be critical for cancer growth and was positively correlated with poor prognosis in cancer patients (40, 41). In terms of metabolism, ETV4 activates PPARγ signaling (42), which directly regulated glycolysis and fatty acid metabolism in cancer cells (43, 44). Similarly, cadherin 3 (*CDH3*) is another highly expressed gene in COAD that encodes P-cadherin and has been linked to poor prognosis in cancer patients and increased glycolysis in cancer cells (45). In LIHC, *PLVAP* was most significantly upregulated compared to normal tissues. This gene has also been found to critically influence cancer development, including facilitating vascular growth (46, 47). Regarding carbohydrate metabolism, we observed an increase in gene sets associated with glycolysis and a decrease in those associated with gluconeogenesis in both COAD and LIHC. These changes in gene expression may indicate a shift towards glycolytic metabolism in these types of cancers. This is consistent with the Warburg effect, a phenomenon in cancer cells in which glycolysis is preferentially used instead of oxidative phosphorylation to generate energy, even in the presence of oxygen.

We investigated the DEGs related to mitochondrial function in COAD and LIHC. Our results showed that genes associated with mitochondrial protein import were significantly upregulated in both COAD and LIHC. Mitochondrial protein import is a crucial component of various physiological processes such as mitochondrial biogenesis, energy metabolism, and maintenance of mitochondrial morphology (48). Recently, the upregulation of mitochondrial protein import-related genes was observed in different cancers (49). Although the exact mechanisms underlying this increase remain unclear, one possible explanation is that the overexpression of these genes may contribute to an increase in mitochondrial biomass (49). Cancer cells rely on glycolysis, which produces less ATP than oxidative phosphorylation, for ATP generation. Therefore, in cancer cells, an increase in mitochondrial biomass may compensate for the reduced ATP generation via glycolysis (50, 51).

Moreover, the present study revealed that in both COAD and LIHC, FAO-associated DEGs were significantly downregulated, whereas the DEGs related to fatty acid synthesis were upregulated. Increased *de novo* lipogenesis (DNL) is a metabolic reprogramming phenomenon in cancer cells. DNL provides a diverse cellular pool of lipid species with various functions, such as membrane structure, ATP synthesis substrate, energy storage, and pro-tumorigenic signaling molecules (52, 53). An increase in DNL is also linked to the activation of oncogenic signaling pathways, such as the PI3K/Akt/mTOR pathway, which is frequently dysregulated in cancer (52). Therefore, further investigation into the role of lipid metabolism in cancer cells is essential for developing new therapeutic strategies targeting cancer-specific metabolic vulnerabilities.

Our results revealed an alteration in the mitochondrial gene *HMGCS2*, which encodes mitochondrial 3-hydroxy-3-methylglutaryl CoA synthase (HMC-CoA synthase), a rate-limiting enzyme for ketogenesis (54). HMGCS2-mediated conversion of Acetoacetyl-CoA to HMG-CoA leads to the production of acetoacetate, which is subsequently converted to β-hydroxybutyric acid, a specific type of ketone body (55). Genome-scale metabolic model analysis showed that HMGCS2 perturbation upregulated the committed steps in the glycolysis pathway and lipid biosynthesis, whereas the committed step in lipid degradation was downregulated. These results suggested that HMGCS2 is important for the metabolic reprogramming of cancer cells.

HMGCS2 is a pivotal enzyme in ketogenesis, a process that is essential for providing alternative energy sources to cells under certain metabolic conditions. Decreased *HMGCS2* expression may lead to reduced ketone body production, which may be a critical factor in the development and progression of GI cancers. The importance of ketogenesis in cancer metabolism is well established, as it contributes to the enhanced energy demands of rapidly proliferating cancer cells. Disruption of ketogenesis can result in the accumulation of reactive oxygen species (ROS) and inflammation, both of which have been linked to tumorigenesis (56). Conversely, ketone supplementation has been shown to exert anti-cancer effects on various types of malignancies. Recently, Ruozheng et al. demonstrated that a ketogenic diet decreased tumor growth and enhanced the anti-cancer effects of immune checkpoint inhibitors in colon cancer (57). Increased ketogenesis due to HMGCS2 overexpression led to similar results. This study revealed that increased ketogenesis suppressed KLF-5 dependent CXCL12 signaling, which is implicated in the growth and metastasis of cancer cells (57). These findings suggest that modulating HMGCS2 activity could be a promising therapeutic strategy for treating colon cancer.

This study had several limitations. First, we assessed metabolic changes based on transcriptome analysis of COAD and LIHC. Further studies using independent datasets and functional experiments are necessary to confirm and extend the findings of the present study. Secondly, this study focused only on early-stage colon and liver cancer samples, and the results may not be applicable to late-stage or other cancer types.

## 5 Conclusions

In conclusion, we identified common and unique transcriptomic signatures associated with COAD and LIHC. These findings suggested that dysregulated lipid metabolism and mitochondrial function play critical roles in the development and progression of these malignancies. Decreased HMGCS2 activity and the related decrease in ketogenesis in GI cancer cells may play crucial roles in the altered energy metabolism observed in these cells. Further investigation into the role of HMGCS2 in GI cancer development and progression could help identify novel therapeutic targets for treating these malignancies.

## Data availability statement

The original contributions presented in the study are included in the article/**Supplementary Material**. Further inquiries can be directed to the corresponding authors.

## Ethics statement

Ethical approval was not required for the study involving humans in accordance with the local legislation and institutional requirements. Written informed consent to participate in this study was not required from the participants or the participants’ legal guardians/next of kin in accordance with the national legislation and the institutional requirements.

## Author contributions

SL, and C-MO contributed to the conceptual design of the project and the experiments described in the manuscript. The experiments were performed by YeMK, YuMK and S-YS. The data were analyzed by YeMK and JJ. The manuscript was written by SL, YeMK, and C-MO. Then, the manuscript was edited and critically evaluated by SL and C-MO. All authors read and approved the final version of the manuscript.

## Glossary

**Table:**

ADAMTS13	ADAM metallopeptidase with thrombospondin type 1 motif 13
ATCC	American type culture collection
ATP	Adenosine triphosphate
BEST4	Bestrophin 4
BP	Biological process
CA7	carbonic anhydrase 7
COAD	colon adenocarcinoma
COL15A1	collagen type XV alpha 1
DEGs	Differentially expressed genes
DMEM	Dulbecco’s Modified Eagle Medium
DNL	*de novo* lipogenesis
ECAR	Extracellular acidification Rate
ETV4	ETS variant transcription factor 4
FAO	fatty acid beta-oxidation
FCCP	Carbonyl cyanide-p-trifluoromethoxyphenylhydrazone
FOXQ1	Forkhead box Q1
G6PC1	Glucose-6-phosphatase 1
G6PC2	Glucose-6-phosphatase
GABRD	gamma-aminobutyric acid type A receptor subunit delta
GI	Gastrointestinal
GLTP	glycolipid transfer protein
GO	Gene ontology
GSEA	Gene set Enrichment Analysis
GSM	Genome-scale metabolic model
HMG-CoA	3-hydroxy-3-methyl-glutaryl-coenzyme
HMGCS2	3-hydroxy-3-methylglutaryl-CoA synthase 2
HR	Hazard ratio
KMO	kynurenine 3-monooxygenase
KRT80	keratin 80
LIHC	liver hepatocellular carcinoma
log2FC	log2foldchange
MAOA	monoamine oxidase A
mTOR	mammalian target of?rapamycin
NES	Normalized enrichment score
OCR	Oxygen Consumption Rate
OIT3	oncoprotein induced transcript 3
OXPHOS	Oxidative phosphorylation
padj	adjusted p-value
PC	Pyruvate kinase
PCA	Principal Component Analysis
PCK1	phosphoenolpyruvate carboxykinase 1
PCK2	phosphoenolpyruvate carboxykinase 2
PFKM	Phosphofructokinase
PFKP	Phosphofructokinase
PI3K	Phosphoinositide 3-kinases
PKM	Pyruvate kinase
PLVAP	plasmalemma vesicle associated protein
ROS	Reactive oxygen species
SDHD	succinate dehydrogenase complex subunit D
siRNA	Small interfering RNA
STAB2	stabilin 2
TCGA	The Cancer Genome Atlas
