# Supplementary material for: Integrative analysis of mitochondrial metabolic reprogramming in early-stage colon and liver cancer
Source: Front Oncol. 2023 Aug 24;13:1218735. doi: 10.3389/fonc.2023.1218735 (PMC10484220; doi:10.3389/fonc.2023.1218735)
Supplement: Supplementary file 1 [file DataSheet_1.pdf]

Supplementary Method

1. Stepwise Genome-scale Metabolic Modeling

1.1 Model generation and flux balance analysis

The HMGCS2 knockout colon model and the HMGCS2 overexpressed colon model were simulated using COBRA Toolbox v.3.0 and the metabolic adjustment minimization method. We generated HMGCS2 knock-out colon models by limiting the lower bounds of the HMGCS2-related reactions (HMR1437, HMR4604, and HMR1573) to nine, while the HMGCS2-overexpressed colon models had upper bounds of 4000 for these three reactions.

1.2 Data Interpretation

If a given reaction has a decreasing flux on the knockout model and an increasing flux on the overexpression model, it was labeled as a 'flux decreasing' reaction. In contrast, if a given reaction has an increasing flux on the knock-out model and a decreasing flux on the overexpression model, then it was classified as a 'flux-increasing' reaction. We emphasize that both the knock-out and overexpression models were used to accurately characterize reaction flux changes, which helped to filter out reaction flux changes from model artifacts and lead to consistent discoveries.

1.3 Data Visualization

We grouped all reaction fluxes by metabolic subsystem. Note that metabolic subsystems were defined using the Human Metabolic Reaction (HMR) database (Mardinoglu, A., Agren, R., Kampf, C. et al. Nat Commun 5, 3083 (2014)). We then counted the number of 'flux increasing' and 'flux decreasing' reactions per metabolic subsystem. The percentage of flux-increasing subsystems in the HMGCS2 knockout colon was obtained by dividing the number of flux-increasing reactions in a given subsystem by the number of reactions in a given

subsystem. Similarly, the percentage of flux decreasing subsystem in HMGCS2 knockout colon was acquired by the number of flux decreasing reactions in a given subsystem divided by the number of reactions in a given subsystem. For example, let the number of reactions in a given metabolic subsystem be N_{total} , the number of flux decreasing reactions in the metabolic subsystem be $N_{decreasing}$, and the number of flux increasing reaction in the metabolic subsystem be $N_{increasing}$. Then, the percent of flux increasing and decreasing subsystem were $N_{increasing}$ / N_{total} and $N_{decreasing}$ / N_{total}, respectively.

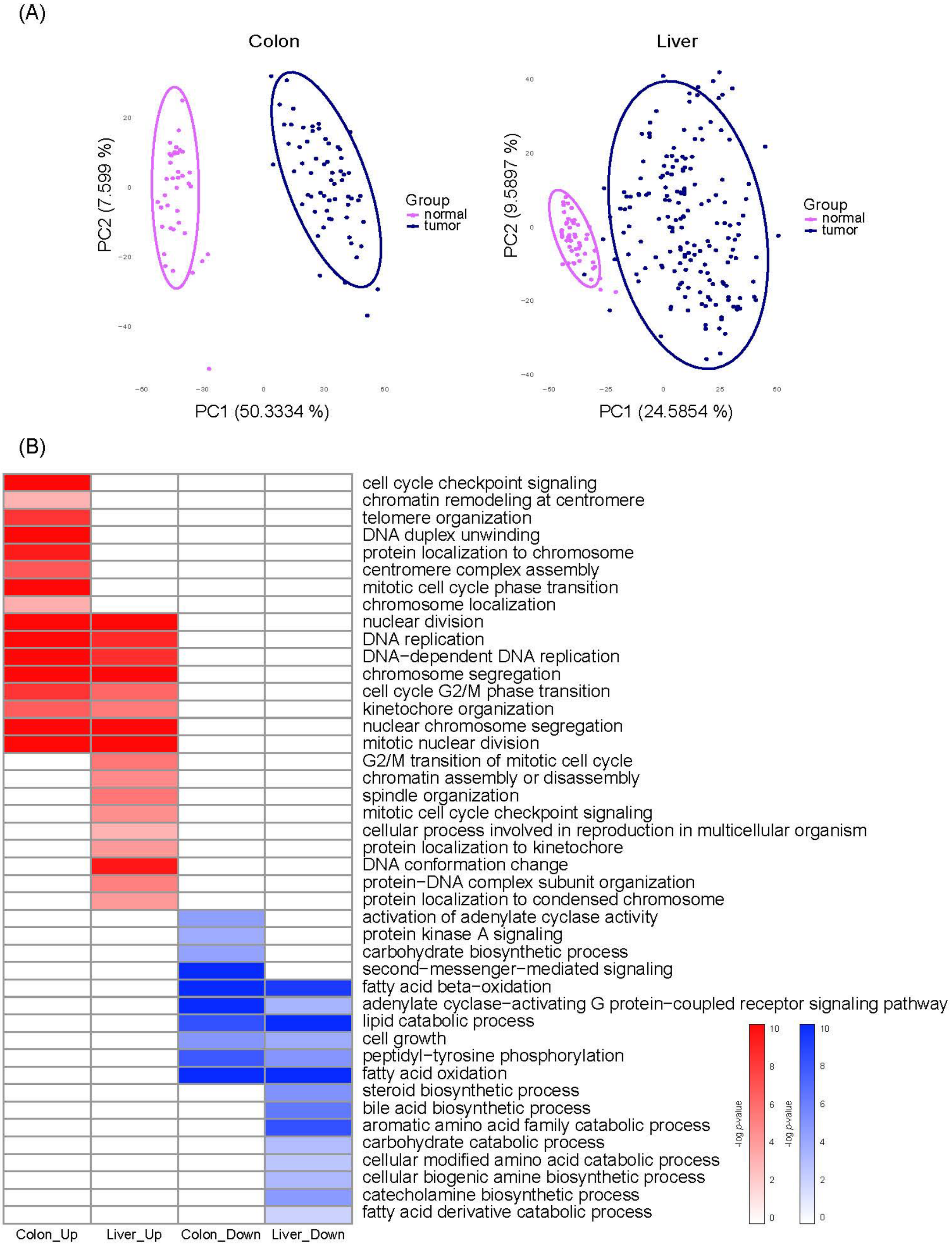

Supplementary Figure 1. Transcriptomic analysis of colon cancer and hepatocellular Carcinoma. (A) Principal component analysis of the RNA-seq data of COAD and LIHC. (B) Heatmap of GSEA enriched pathways from DEGs of COAD and LIHC. DEG: differentially expressed gene, COAD: colon adenocarcinoma, LIHC: liver hepatocellular carcinoma, GSEA: gene set enrichment analysis.

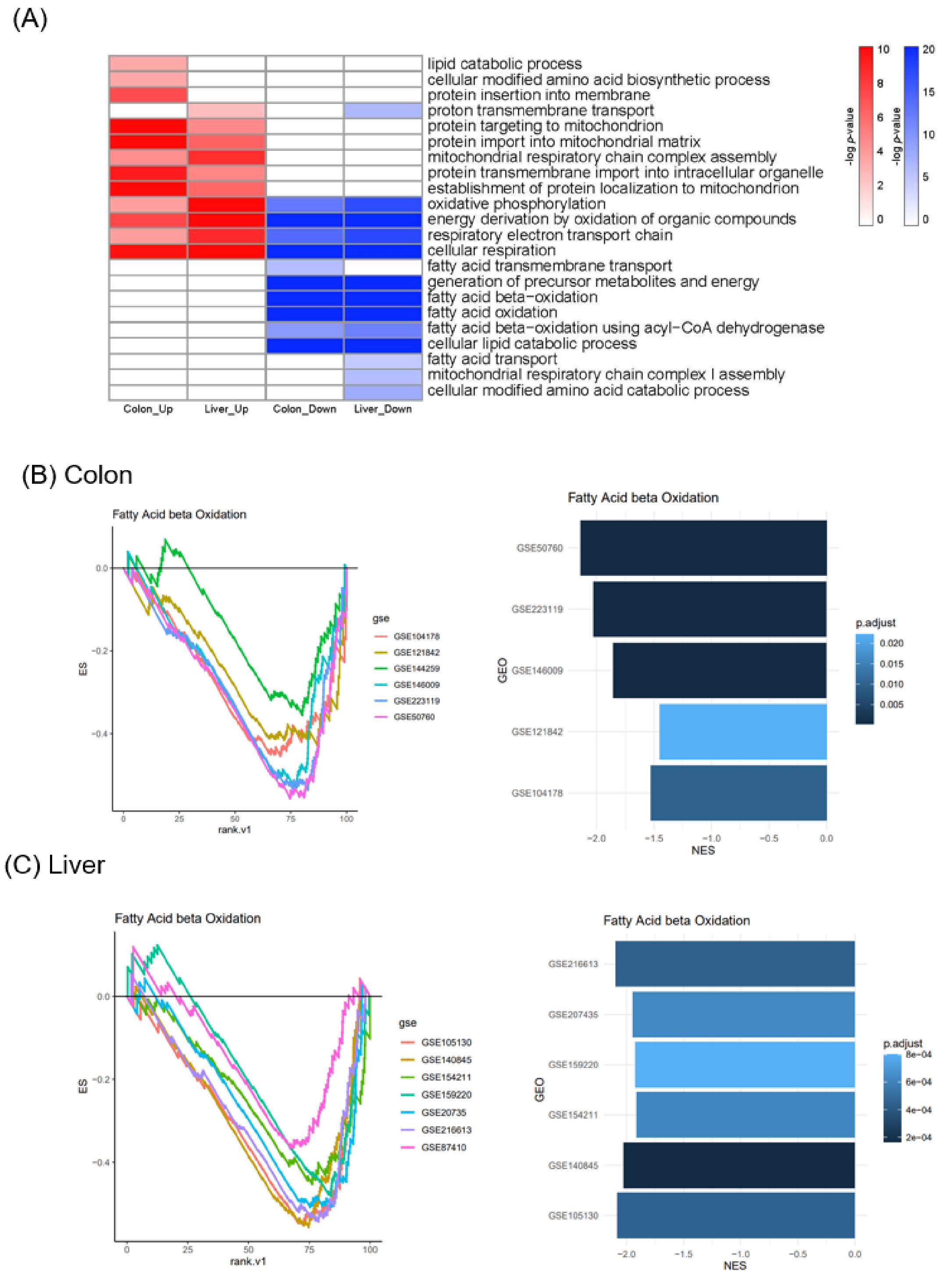

Supplementary Figure 2. GSEA enriched pathways from mitochondrial DEGS of COAD and LIHC. (A) Heatmap of GSEA enriched pathways. GSEA plot and bar related fatty acid oxidation in publicly available colon cancer (B) and liver cancer (C) datasets. DEG: differentially expressed gene, COAD: colon adenocarcinoma, LIHC: liver hepatocellular carcinoma, GSEA: gene set enrichment analysis.

(A) Colon

(B) Liver

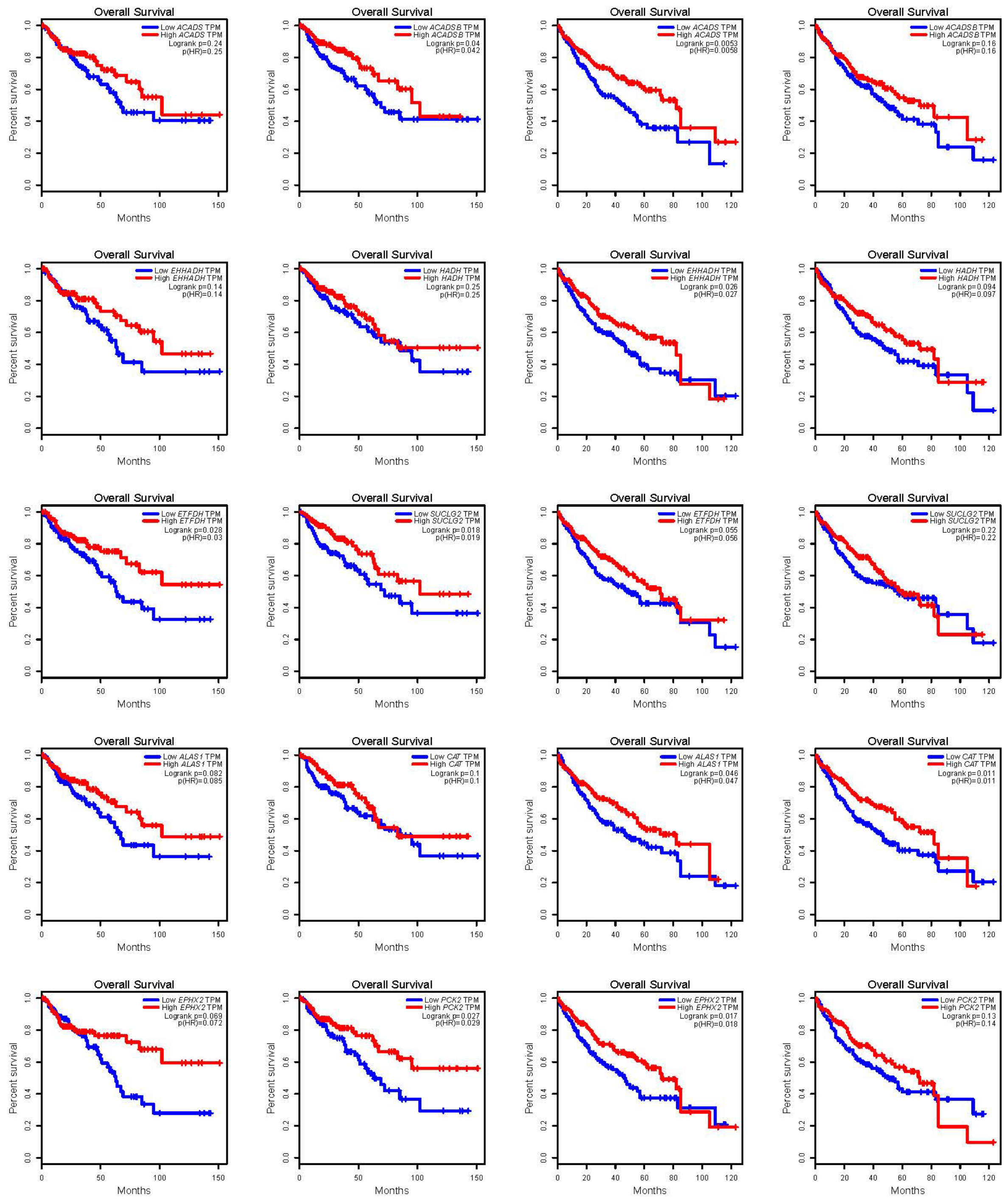

Supplementary Figure 3. Overall survival according to expressions of genes related to fatty acid oxidation in COAD (A) and LIHC (B).

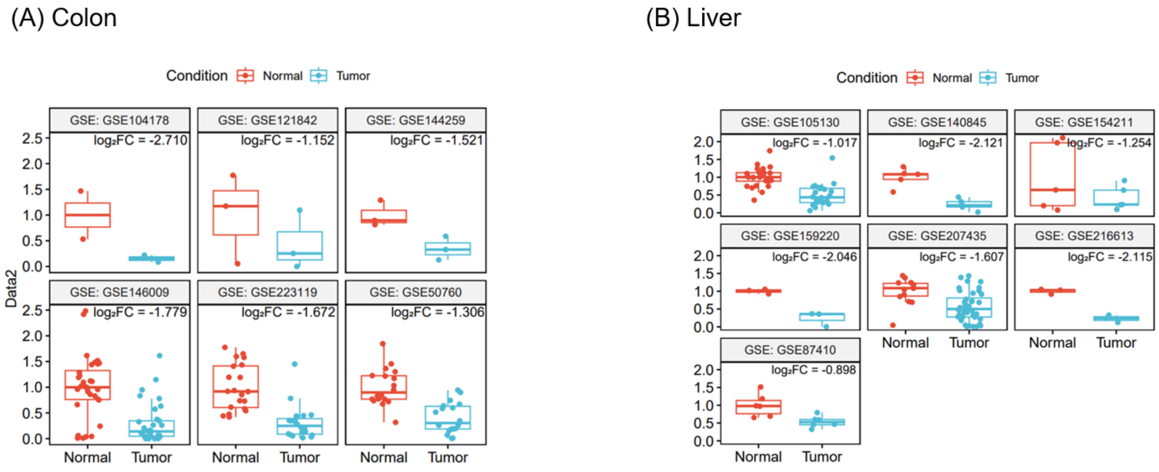

Supplementary Figure 4. HMGCS2 expression in colon cancer and liver cancer.

HMGCS2 expressions in public colon cancer datasets (A) and public liver cancer datasets (B).

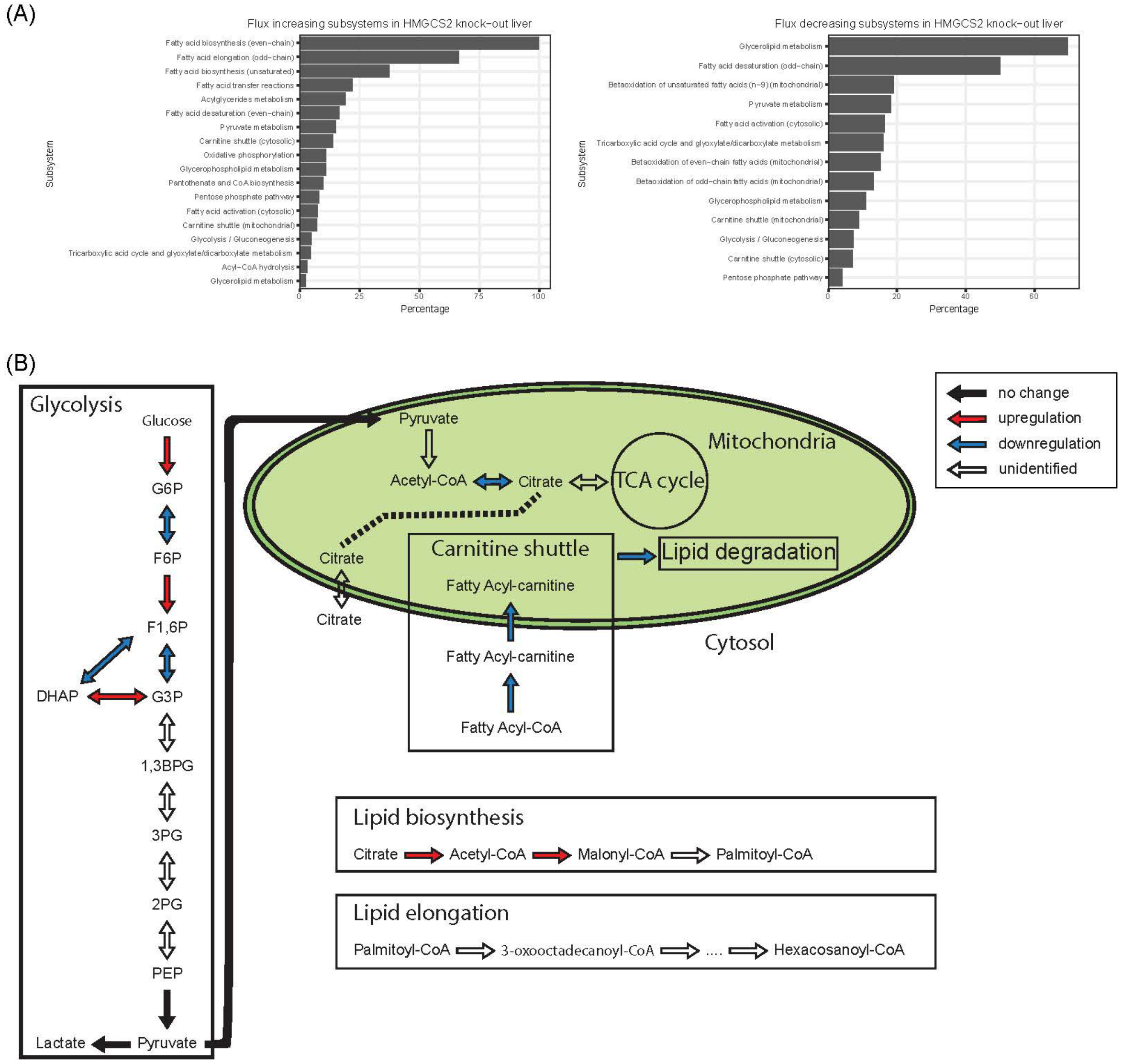

Supplementary Figure 5. HMGCS2-driven metabolic flux in liver. (A) Bar plots of predicted increasing and decreasing subsystems according to *HMGCS2* knock-out in liver using genome-scale metabolic model. (B) Schematic overview of the metabolic flux according to *HMGCS2* knock-out in liver in the genome-scale metabolic model.

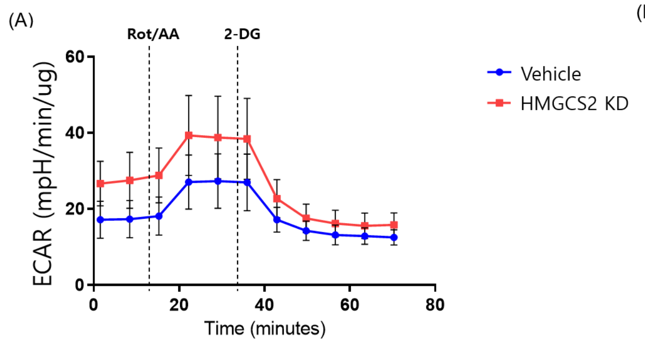

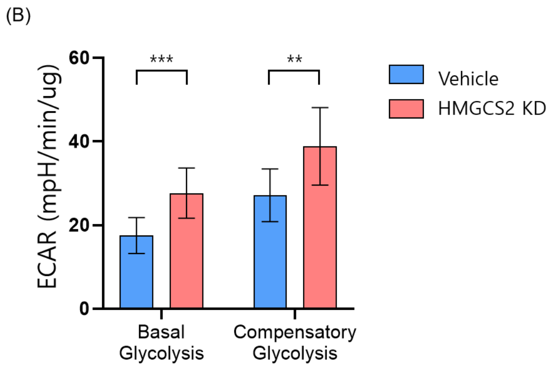

Supplementary Figure 6. Extracellular Acidification Rate (ECAR) of Caco-2 cell.
